# Optimizing the creation of base populations for aquaculture breeding programs using phenotypic and genomic data and its consequences on genetic progress

**DOI:** 10.3389/fgene.2014.00414

**Published:** 2014-11-25

**Authors:** Jesús Fernández, Miguel Á. Toro, Anna K. Sonesson, Beatriz Villanueva

**Affiliations:** ^1^Departamento de Mejora Genética Animal, INIAMadrid, Spain; ^2^Departamento de Producción Animal, ETSI Agrónomos, Universidad Politécnica de MadridMadrid, Spain; ^3^NofimaÅs, Norway

**Keywords:** base populations, aquaculture breeding programs, SNP markers, optimal contributions, fish genomics

## Abstract

The success of an aquaculture breeding program critically depends on the way in which the base population of breeders is constructed since all the genetic variability for the traits included originally in the breeding goal as well as those to be included in the future is contained in the initial founders. Traditionally, base populations were created from a number of wild strains by sampling equal numbers from each strain. However, for some aquaculture species improved strains are already available and, therefore, mean phenotypic values for economically important traits can be used as a criterion to optimize the sampling when creating base populations. Also, the increasing availability of genome-wide genotype information in aquaculture species could help to refine the estimation of relationships within and between candidate strains and, thus, to optimize the percentage of individuals to be sampled from each strain. This study explores the advantages of using phenotypic and genome-wide information when constructing base populations for aquaculture breeding programs in terms of initial and subsequent trait performance and genetic diversity level. Results show that a compromise solution between diversity and performance can be found when creating base populations. Up to 6% higher levels of phenotypic performance can be achieved at the same level of global diversity in the base population by optimizing the selection of breeders instead of sampling equal numbers from each strain. The higher performance observed in the base population persisted during 10 generations of phenotypic selection applied in the subsequent breeding program.

## Introduction

The success of an aquaculture breeding program critically depends on the way in which the base population of breeders is constructed (Hayes et al., 2006; Holtsmark et al., 2006, 2008a,b). In particular, all the genetic variability that can be used for selection on the traits initially included in the breeding objective is that found in the original breeders. Also, the decisions taken when creating the base population will have consequences on the genetic progress for any other additional trait that may be part of future breeding goals, whatever production or fitness related traits.

Traditionally, base populations in aquaculture breeding programs were created from wild or from domesticated strains not subjected to any formal selection program (see Gjedrem et al., 1991, for details on the formation of the base population in the Norwegian breeding program for Atlantic salmon and Eknath et al., 1993, 1998, for details on the formation of the base population in the GIFT breeding program for tilapia). In this context, Holtsmark et al. (2006, 2008a) investigated through computer simulation the effects of the number of strains contributing to the base population and the mating strategy (within and across strains) on the genetic gain achieved in the subsequent selection program. These studies assumed wild populations and no knowledge of the genetic structure (i.e., within and between strain diversity) or phenotypic levels for the trait of interest when setting up the base population. Consequently, the simulated strategy was randomly sampling the same number of individuals from each strain to form the base population. However, the solution leading to the highest level of diversity and acceptable levels of phenotypic performance when starting the breeding program surely will depart from equal proportions.

In the absence of genealogies (which could be the case even in improved commercial strains), molecular markers can be used to estimate relationships between and within populations. Hayes et al. (2006) compared random with molecular-based optimized selection of breeders in terms of the genetic variance captured for growth and for two disease traits in Atlantic salmon. However, no considerations about the phenotypic level of candidates were made when sampling the breeders and the number of markers they used was scarce (237 AFLP).

The present availability of large panels of SNPs makes marker diversity more informative on the global genetic variability in the genome than diversity computed from genealogical data (Gómez-Romano et al., 2013). Nowadays, dense SNP chips have been already developed for Atlantic salmon (130K, Houston et al., 2014), rainbow trout (57K, Palti et al., 2014), catfish (250K, Liu et al., 2014) and carp (250K, Xu et al., 2014). This development of an increasing number of markers for aquaculture species will make it possible in a near future to have accurate measures of genetic relationships between and within strains. In this new scenario, the optimal number of individuals to be sampled from each strain could be calculated in a similar way as when optimizing the construction of mixed populations from different origins in conservation programs aimed at capturing the highest levels of genetic diversity (Eding and Meuwissen, 2001; Caballero and Toro, 2002; Eding et al., 2002).

Nowadays, for some aquaculture species already genetically improved strains are available. The use of improved rather than wild strains when creating base populations would allow the new breeding program to begin from higher phenotypic levels for the trait of interest, making it competitive from the start. The necessity of taking into account the phenotypic value of each candidate strain is even clearer when searching for new breeders to be included in an already established breeding program whose genetic variability has been greatly reduced. The increase in genetic variability should be achieved without compromising the gain in performance previously obtained through artificial selection.

The objective of this paper was to study, using computer simulations, the consequences of using genome-wide molecular information to compute genetic relationships and phenotypic records to optimize the sampling of individuals from different strains when creating base populations in aquaculture breeding programs. Different scenarios varying in the type of strains (wild or commercial), the degree of relationships within and between strains and the level of information (individual or strain information) were considered for generic aquaculture population designs. Results were compared in terms of phenotypic level for the trait of interest and the diversity achieved in the base population and in subsequent generations of selection.

## Materials and methods

### Genome structure

Diploid individuals were simulated with a genome comprising 20 chromosomes of 1 Morgan each. This genome length could be representative of the genomic architecture of the most cultivated fish species in aquaculture (which have genomes ranging from 10 to 35 Morgans). Each chromosome carried 25,000 neutral biallelic loci that will be referred to as “non-marker loci” thereafter. Additionally, 5–5000 biallelic markers per chromosome (equivalent to SNPs) were simulated interspersed with the neutral loci. Therefore, the total number of available markers ranged from 100 to 100,000 and the density of markers ranged from 5 to 5000 markers/M. All loci were evenly spaced within each chromosome. Markers were used to optimize the contributions of the different strains to the base population while non-marker loci (i.e., the 25,000 neutral loci per chromosome) were used to monitor the effect on genetic diversity of the different strategies evaluated (see below). With dense marker panels we expect that most non-marker loci in the genome will be in linkage disequilibrium (LD) with at least some of the markers and, thus, managing diversity using marker genotypes will lead to the maintenance of diversity in the rest of the genome.

### Generation of candidates

The creation of the candidates to contribute to the base population followed a two-step process. First, a large population in mutation-drift equilibrium was generated. Then, individuals were sampled from this population to form different strains which were allowed to diverge for several generations before being available for the selection of the individuals to be included in the base population.

#### Equilibrium population

First, to obtain a realistic pattern of linkage disequilibrium, a large population (*N* = 1000 with equal number of males and females) under random selection was simulated for 1000 discrete generations. Each generation, sires and dams were sampled with replacement and population size was kept constant across generations. Initially, genotypes for the individuals were assigned at random independently for each locus (markers and non-markers). Consequently, the initial allelic frequencies were 0.5 for all loci and Hardy-Weinberg and linkage equilibria existed within and between loci, respectively. During this period, mutation was allowed to occur throughout the genome. The mutation rate per locus and generation was μ = 2.5 × 10^−3^ for both types of loci (marker and non-marker). The number of new mutations simulated at every generation was sampled from a Poisson distribution with mean 2*Nn*_*c*_μ*n*_*l*_ where *n*_*c*_ is the number of chromosomes and *n*_*l*_ is the total number of loci (markers and non-markers) per chromosome. Mutations were then randomly distributed across individuals, chromosomes and loci, switching allele 0 to allele 1 and vice versa. When generating the gametes, the number of crossovers per chromosome was drawn from a Poisson distribution with mean equal to 1. Crossovers were randomly distributed without interference. At the end of the process the expected heterozygosity of the population had already reached an equilibrium value.

#### Creation of strains

The second step of the process consisted in randomly sampling 10 different groups of individuals (mimicking 10 different strains) from the population at equilibrium. At this step, a quantitative trait with phenotypic mean (μ), initial phenotypic variance [*V*_*P*(0)_] and heritability [*h*^2^_(0)_] of 100, 30, and 0.4, respectively, was defined. The trait, measured in both sexes, was controlled by 1000 additive loci (thereafter called selective loci). These selective loci were chosen at random from the previously simulated loci (markers and non-markers). The additive effect of locus *i* (*a*_*i*_) was sampled from a normal distribution with mean 0 and variance *V*_*A*_(0)/[2*p*(1–*p*)*n*_*sel*_], where *V*_*A*(0)_ is the initial additive variance (*h*^2^_(0)_*V*_*P*_(0) = 12), *p* is the average frequency across selective loci and *n*_*sel*_ is the number of selective loci (i.e., 1000). Note that, in this way, the expected additive variance summed over all loci equals *V*_*A*_(0), assuming that covariances between loci generated by LD are negligible. The phenotypic value for a particular individual *j* was obtained as $P_{j}=\mu +\sum_{i\;=\;1}^{nsel}x_{i}a_{i}+e_{j}$, where *x*_*i*_ is an indicator variable that takes values 1, 0 or −1 for homozygous 11, heterozygous or homozygous 00, respectively, and *e*_*j*_ is the individual environmental deviation that was sampled from a normal distribution with mean 0 and variance *V*_*E*_ = *V*_*A*_(0)(1–*h*^2^_(0)_)/*h*^2^_(0)_. The environmental variance (*V*_*E*_) was initially calculated for each replicate and strain in order to assure that all started with the same *h*^2^_(0)_ value and was kept constant across generations.

Once the strains were created, they were allowed to diverge during 20 discrete generations (with constant population size) under three different regimes: (i) random selection; (ii) artificial selection with different selection pressures in order to mimic already improved strains; and (iii) stabilizing selection with different optima in order to mimic wild strains with local adaptations in nature. Four different scenarios varying in the type of strains they included were then defined:

1. Drift Scenario. All strains were created under random selection but they differed in size (from 10 to 40 individuals, half of each sex) in order to simulate different degrees of genetic drift. Smaller sizes will lead to lower within strain diversity but higher differentiation between strains.
2. Selection Scenario. All strains were created under artificial directional selection for the simulated trait. They had all the same size (20 males and 20 females) but differed in the strength of the selection imposed. The highest selection pressure corresponded to selecting the two males and the two females with the highest phenotypic values for the trait, and the lowest pressure corresponded to selecting five males and five females.
3. Stabilizing Scenario. All strains were created under stabilizing selection for the simulated trait. The probability of survival of an individual with phenotypic value *P* was modeled using a Gaussian distribution (Turelli, 1984; Bürger et al., 1989):
  $$W(P)=\exp\left(-\frac{{\left(P-P_{opt}\right)}^{2}}{2\omega^{2}}\right)$$
  where *P*_*opt*_ is the optimum phenotype in a particular environment and ω^2^ is an inverse measure of the strength of stabilizing selection. For some strains *P*_*opt*_ was set to the original mean (100), for others the optimum was set to a lower value (90) and for others the optimum was set to a higher value (110). Selection pressure was relatively strong (ω^2^ = 5) and population size was equal in all strains (20 males and 20 females).
4. Mixed Scenario. The set comprised strains of the three types described above (i.e., randomly selected, under directional selection and under stabilizing selection).

Table 1 summarizes the specific parameters used when creating the 10 strains under each scenario. The sizes of the strains simulated during the 20 generations were rather small in order to force a rapid divergence between them and, thus, real differences in relatedness and phenotypic levels at the end of the differentiation stage. After this period, subpopulations were expanded in order to have a large number of candidates to form the base population. Specifically, the population size of each strain increased to 50 males and 50 females in a single generation of random selection and mating.

**Table 1:** **Parameters used to generate each strain for the four different scenarios simulated**.

	**Drift**	**Selection**		**Stabilizing**		**Mixed**		
**Strains**	**Size**	**Size**	**Numbers selected**	**Size**	**Optimum phenotype**	**Size**	**Optimum phenotype**	**Numbers selected**
1	5	20	2	10	90	5	–	–
2	5	20	2	10	90	10	–	–
3	5	20	3	10	90	10	–	–
4	10	20	3	10	100	20	–	–
5	10	20	4	10	100	10	90	–
6	10	20	4	10	100	10	100	–
7	20	20	4	10	100	10	110	–
8	20	20	5	10	110	10	–	5
9	20	20	5	10	110	10	–	3
10	20	20	5	10	110	10	–	1

Size and Numbers selected refer to the number of individuals per sex.

### Foundation of the base population

From the 1000 available candidates (50 males and 50 females from each strain) for each particular scenario, base populations were constructed by selecting 100 males and 100 females following different strategies: (i) Taking at random equal numbers of individuals from each strain (strategy E); (ii) Determining optimal strain proportions for maximizing the expected heterozygosity (*H*_*e*_) calculated from the mean coancestry values within and between strains (strategy MC); (iii) Determining optimal strain proportions for maximizing the mean (strain) phenotypic value with a restriction on coancestry (strategy MP); (iv) as in (ii) but using individual relationships instead of strain means (strategy IC); and (v) as in (iii) but maximizing individual values of the selected individuals instead of strain means (strategy IP). Note that, in these abbreviations, M and I stand for mean and individual information, respectively, C indicates that the objective is just to minimize coancestry (i.e., maintain high levels of *H*_*e*_) and P indicates that the objective is to create base populations with a high phenotypic level.

Strategy E is equivalent to that used by Holtsmark et al. (2006, 2008a,b) and provides a reference point for comparisons. Strategy MC followed the methodology presented by Eding and Meuwissen (2001) and Caballero and Toro (2002). The particular objective function to minimize was $\sum_{i\;=\;1}^{N_{s}}\sum_{j\;=\;1}^{N_{s}}c_{i}c_{j}f_{ij}$, where *N*_*s*_ is the number of strains, *c*_*i*_ is the proportion of individuals to be sampled from strain *i* and *f*_*ij*_ is the mean coancestry coefficient between strains *i* and *j* calculated from marker genotypes. Contributions were forced to be in the interval [0,1] and to sum up to 1.

Strategy MP searched for the solution that maximized the objective function $\sum_{i\;=\;1}^{N_{s}}c_{i}P_{i}$, where *P*_*i*_ is the mean phenotype of strain *i* but imposing the restriction $\sum_{i\;=\;1}^{N_{s}}\sum_{j\;=\;1}^{N_{s}}c_{i}c_{j}f_{ij}\leq C_{E}$, where *C*_*E*_ is the mean coancestry of the base population obtained under strategy E. The restrictions imposed in MC were also applied to MP. In the two strategies relying on strain mean values (MC and MP) the actual number of individuals to be sampled from each strain was obtained by multiplying *c*_*i*_ by the total number of individuals to be selected and rounding to the nearest even integer. This procedure was implemented to ensure that half of the individuals from each strain were males and half females.

The objective under strategies IC and IP was to minimize $\sum_{i\;=\;1}^{N}\sum_{j\;=\;1}^{N}x_{i}x_{j}f_{ij}$ and to maximize $\sum_{i\;=\;1}^{N}x_{i}P_{i}$, respectively. Here *N* is the total number of candidates (i.e., all individuals from every strain), *f*_*ij*_ is the coancestry between individuals *i* and *j*, *P*_*i*_ is the phenotype of individual *i* and *x*_*i*_ is an indicator variable that takes a value of 1 if individual *i* is to be selected and 0 otherwise. The sum of *x*'s for males and females was forced to be equal to the number of individuals to be selected for creating the base population (i.e., 100 of each sex). Strategy IP also included a restriction to guarantee that solutions had a global coancestry lower or equal than that obtained under E strategy. All the optimizations were performed using “simulated annealing” algorithms (Kirkpatrick et al., 1983).

It must be emphasized that in strategies MC and MP the output of the optimizations is the proportion of individuals to be taken from each strain but the specific individuals are sampled at random. This could be the situation when prior knowledge of the mean genetic relationship or performance of the strains is available but individual information for the candidates is not. In strategies IC and IP the outputs are the particular individuals to be selected as we assume that their genotypes and phenotypic values are known.

As described above, restrictions imposed under strategies MP and IP were set to the level of coancestry (calculated on the markers) obtained under strategy E. Thus, these three strategies could be compared in terms of phenotypic level at the same diversity level.

### Artificial selection

To explore the consequences of each strategy for creating the base population on genetic gain and diversity in the subsequent breeding programme, 10 generations of artificial selection were simulated for each combination of marker density, type of available strains and strategy. At generation *t* = 0 (i.e., base population), founders were mated at random to form 100 families and 10 offspring were obtained from each couple. Consequently, 1000 individuals were available as candidates for selection. The 100 males and 100 females with the highest phenotypic value for the simulated trait were selected to produce generation *t* + 1 (i.e., phenotypic truncation selection was conducted). Selected individuals were mated at random and, again, 10 offspring were generated from each couple. Therefore, the proportion of selected individuals was 20% that corresponds to a selection intensity of 1.4.

### Variables for comparison

In the base population, comparisons between strategies were made in terms of the mean phenotypic value (*P*) and *H*_*e*_ of the group of selected individuals. Note that *H*_*e*_, calculated on the non-marker loci, is a measure of the genetic variation of the population and its ability to adapt to new environments. The contributions of strains to the base population, measured as the proportion of breeders selected from each of the strains, was also considered in the comparisons.

In the artificial selection step, the strategies were also compared in terms of mean breeding value (*BV*) and additive variance (*V*_*A*_) for the target trait, genealogical inbreeding (*F*) and coancestry (*f*) coefficients and rates of gain, inbreeding (Δ*F*) and coancestry (Δ*f*). For the computation of inbreeding and coancestry, founders in the base population were assumed to be unrelated and non-inbred. In all scenarios, values presented are averages of 100 replicates.

## Results

### Contributions of strains to the base population

The proportional contribution of each available strain to the base population is shown in Table 2 for the most extreme marker densities, the four scenarios and the five strategies simulated. In general, the observed patterns were the same when using high or low density of markers. Particular differences in performance due to marker density are highlighted below.

**Table 2:** **Contributions of each strain to the base population (in percentage) under different strategies to select individuals for the base population for the four scenarios considered and for different number of markers**.

**Markers**		**Drift**					**Selection**				**Stabilizing**				**Mixed**			
		**E**	**MC**	**MP**	**IC**	**IP**	**MC**	**MP**	**IC**	**IP**	**MC**	**MP**	**IC**	**IP**	**MC**	**MP**	**IC**	**IP**
100	1	10.0	−3.3	−3.6	−3.6	−2.9	−1.5	−3.8	−1.6	−4.3	0.6	−4.1	−0.1	−7.6	−0.9	−2.9	−1.2	−4.1
	2	10.0	−5.0	−4.0	−4.4	−1.8	−1.8	−4.5	−1.7	−5.0	−0.7	−5.1	−0.6	−7.6	4.0	1.0	3.3	−1.5
	3	10.0	−5.1	−4.4	−4.5	−2.1	0.1	−0.2	−0.3	−0.5	−1.1	−5.4	−0.7	−7.8	2.1	−1.2	2.5	−2.5
	4	10.0	−0.5	0.6	−0.6	1.0	−0.9	−1.5	−0.7	−1.2	1.5	1.7	0.8	0.4	12.4	5.4	9.8	0.1
	5	10.0	−2.6	−1.4	−1.6	0.5	0.5	1.6	0.1	1.3	0.7	0.7	0.9	0.2	−2.3	−7.7	−1.7	−8.9
	6	10.0	−2.2	−3.1	−1.5	−1.9	−0.4	0.1	0.0	0.8	0.3	−0.1	0.6	−0.6	−1.9	−3.8	−1.1	−4.2
	7	10.0	4.6	4.3	3.9	2.0	−0.3	0.7	0.1	1.3	−0.5	−0.7	−0.1	−0.7	−2.7	−0.4	−2.0	1.0
	8	10.0	4.2	3.8	3.9	2.1	2.0	3.2	1.3	2.7	−0.3	4.3	−0.7	7.7	−1.7	7.0	−1.0	10.9
	9	10.0	5.8	5.3	5.1	1.9	0.0	1.0	0.5	1.6	−0.9	3.8	−0.5	7.6	−4.1	3.8	−3.5	8.3
	10	10.0	4.1	2.3	3.5	1.1	2.3	3.3	2.3	3.4	0.4	5.0	0.4	8.5	−4.9	−1.2	−5.1	0.8
		*0.0*	*16.2*	*12.7*	*12.6*	*3.4*	*1.6*	*6.2*	*1.3*	*7.1*	*0.6*	*13.4*	*0.4*	*36.7*	*23.6*	*18.0*	*16.2*	*31.1*
100,000	1	10.0	−4.0	−3.8	−4.8	−3.3	−2.0	−3.5	−2.0	−3.0	0.4	−1.3	−0.4	−2.0	−1.2	−2.8	−2.0	−3.2
	2	10.0	−4.9	−4.7	−4.8	−3.5	−2.1	−3.6	−1.9	−3.0	−0.6	−2.3	−0.5	−2.2	3.3	0.9	2.7	0.4
	3	10.0	−5.0	−4.8	−4.7	−3.5	−0.9	−0.7	−1.0	−0.9	−0.7	−2.4	−0.4	−2.1	2.5	0.3	2.8	0.6
	4	10.0	−0.9	−0.5	−1.5	−0.3	−1.2	−1.1	−1.0	−0.8	1.3	1.3	0.7	0.6	12.4	8.3	12.2	6.8
	5	10.0	−1.8	−1.3	−1.6	−0.2	1.0	1.8	0.4	0.9	0.4	0.4	0.7	0.6	−2.4	−7.4	−2.2	−6.5
	6	10.0	−1.8	−1.2	−1.6	−0.3	0.0	0.6	0.3	0.8	0.5	0.4	0.7	0.7	−1.6	−3.2	−1.3	−2.6
	7	10.0	4.3	2.5	4.6	1.7	−0.1	0.2	0.2	0.4	0.2	0.3	0.6	0.5	−2.6	−0.6	−2.4	−0.3
	8	10.0	5.2	6.0	4.9	4.0	2.4	2.8	1.7	1.9	−0.2	1.6	−0.4	1.3	−1.4	5.4	−1.2	5.3
	9	10.0	4.5	3.8	4.8	2.7	1.4	1.7	1.7	1.8	−0.6	1.0	−0.4	1.3	−3.9	1.6	−3.7	1.8
	10	10.0	4.5	3.9	4.8	2.8	1.4	1.8	1.6	2.0	−0.7	0.9	−0.5	1.2	−5.1	−2.4	−5.1	−2.2
		*0.0*	*15.9*	*13.6*	*16.7*	*7.0*	*2.1*	*4.4*	*1.8*	*3.2*	*0.4*	*2.0*	*0.3*	*1.9*	*23.0*	*18.0*	*22.1*	*14.1*

Contributions for strategies MC, MP, IC, and IP are given as deviations from those of strategy, E. The variance of contributions across strains is given in italics. E, equal numbers from each strain; MC, minimization of coancestry based on mean strain values; MP, maximization of phenotype with restriction on coancestry based on mean strain values; IC, minimization of coancestry based on individual values; IP, maximization of phenotype with restriction on coancestry based on individual values.

In the Drift scenario no meaningful differences in contributions were observed between strategies MC, MP, and IC for a particular strain. This was due to the fact that no selection on the quantitative trait was exerted during the generation of the strains and, therefore, the phenotypic mean was equal to the initial value before the divergence period (i.e., 100) for all strains. However, differences arose between strains. The higher the population size of a particular strain the higher was its contribution. Strains with a small size (strains 1, 2, and 3) had the lowest contributions due to the large loss of genetic diversity during the divergence period. Therefore, these strains were less useful for increasing the amount of diversity stored in the synthetic base population. The opposite happened with large size strains (7, 8, 9, and 10) that contributed more than 14% each to the base population (see Drift scenario in Table 2). The lowest variance of contributions between strains (beyond strategy E) was found under strategy IP. This could be explained by the fact that strong drift in small populations could result in the existence of individuals with extreme high phenotype. Thus, as the main objective of strategy IP is achieving a high phenotypic level in the base population, it would be worthy to keep individuals not only from large but also from small strains. In this situation a high phenotypic level can be obtaining without reducing too much the diversity maintained.

In the Selection scenario, the variance of contributions across strains was much lower than in the Drift scenario (1.3–6.2 vs. 12.6–16.7; Table 2) except for strategy IP with low marker density. This is a consequence of the greater uniformity between strains, at least for the genetic variability of the trait. In the Selection scenario all strains had been under directional selection for the same trait and all had the same size. The only difference between strains was the strength of selection. Those under the weakest selection pressure (8, 9, and 10) had a higher effective population size (*N*_*e*_), maintained higher levels of genetic diversity and, thus, in average contribute more to the base population. Strains subjected to a strong selection pressure were those with the lowest contributions, even under the strategies directed to keep high levels of trait performance (see strains 1 and 2, Selection scenario in Table 2). This is due to the relative long time period since the separation of the strains (20 generations). The small *N*_*e*_ induced by the selection pressure erodes rapidly not only neutral variability but also the genetic variance of the trait. Therefore, strains 1 and 2 reached a selection limit before the rest and presented lower phenotypic mean values at the time the base population was created (data not shown). Consequently, they cannot contribute much to diversity nor to trait value either.

In the Stabilizing scenario, when the criteria for choosing individuals to constitute the base population included considerations on the phenotypic performance (i.e., strategies MP and IP) the contribution of a strain was proportional to its phenotypic mean (Table 2). Contrarily, contributions were almost equalized when the only concern was to keep the highest levels of diversity (strategies MC and IC), given that all strains had an identical population size and a similar selection pressure in the divergence period. An interesting observation was that the variance of contributions across strains greatly decreased for strategies MP and IP when a large panel of SNPs (100,000) was used (see lower section of Table 2). With dense genotyping, diversity at selective loci is tightly linked to neutral diversity and, thus, groups of individuals with high phenotype will also have low diversity at the markers. Therefore, optimal solutions include the selection of fewer individuals from the same high performance strain to cope with the restriction on genetic diversity. The lower diversity of high performance groups of individuals is not detected with sparse marker coverage as diversity at selective loci is loosely linked to neutral diversity.

The Mixed scenario included all kind of strains and, thus, the performance was somehow more complex. Notwithstanding, the general patterns highlighted before (i.e., dependence of contributions on the historical size, the intensity of selection and the optimal phenotypic value, respectively) are still observable (see right part of Table 2). A particular observation was that the contribution of strains adapted to a low phenotypic value (i.e., strains created under stabilizing selection with a low optimum) was no longer required even with massive genotyping. Also, it was observed that under strategies MP and IP the low diversity of high performance individuals may be compensated for the high diversity of the “drift” strains with reasonable phenotypic levels.

### Diversity and phenotypic level in the base population

The mean phenotypic value for the trait of interest and the genetic diversity captured in the group of individuals conforming the base population are presented in Table 3. It must be pointed out that all strategies except E are explicitly concerned with the maintenance of diversity. However, only MP and IP strategies included considerations about the phenotypic level in the objective function to optimize.

**Table 3:** **Average phenotypic value and expected heterozygosity (in percentage) under different strategies to select individuals for the base population for the four scenarios considered and for different number of markers (*n*_*m*_)**.

**Scenario**	***n*_*m*_**	**Phenotypic value**					**Expected heterozygosity**				
		**E**	**MC**	**MP**	**IC**	**IP**	**E**	**MC**	**MP**	**IC**	**IP**
Drift	100	99.93	99.88	101.44	99.97	106.14	46.21	46.23	45.63	46.32	45.77
	1000	99.81	99.73	100.98	99.76	105.78	46.22	46.49	46.11	46.51	46.14
	100,000	100.06	100.00	101.29	99.98	105.97	46.18	46.48	46.12	46.60	46.19
Selection	100	129.02	129.12	130.45	129.09	134.41	44.54	44.17	43.69	44.26	43.64
	1000	127.96	128.11	128.82	128.07	132.81	44.55	44.56	44.41	44.58	44.36
	100,000	128.26	128.37	129.01	128.04	132.87	44.51	44.57	44.47	44.67	44.51
Stabilizing	100	100.00	99.95	102.70	100.01	107.94	45.14	44.78	44.37	44.87	43.81
	1000	99.99	100.03	102.65	100.08	107.91	45.13	44.75	44.33	44.84	43.80
	100,000	99.02	98.95	99.97	98.99	104.15	44.66	45.00	44.62	45.00	44.65
Mixed	100	106.27	104.07	109.49	104.32	115.50	45.24	45.42	44.70	45.49	43.93
	1000	105.95	103.94	108.15	104.13	113.05	45.34	45.71	45.27	45.74	45.08
	100,000	106.08	103.97	108.12	103.97	112.58	45.33	45.72	45.31	45.84	45.33

E, equal numbers from each strain; MC, minimization of coancestry based on strains values; MP, maximization of phenotype with restriction on coancestry based on strains values; IC, minimization of coancestry based on individual values; IP, maximization of phenotype with restriction on coancestry based on individual values. Standard errors of phenotypic value ranged from 0.13 to 0.25 and those for expected heterozygosity were lower than 0.01%.

Using strategy IP with a large number of markers in the Mixed scenario led to a base population with mean phenotypic value for the trait 7% higher than under strategy E at the same diversity. The advantage of strategy IP over E in other scenarios ranged from about 4% (Selection scenario) to 6% (Drift scenario), being proportional to the degree of differentiation between strains. This is a logical result if we realize that the margin for improvement with unequal contributions was lower when strains were more similar (for example in the Selection scenario). When relying on the information of few markers, IP led to higher trait means than with dense genotyping (left part of Table 3) because diversity at selective loci and at the markers was more loosely linked. Then, groups of individuals with high phenotypic mean for the trait can be found also showing high levels of diversity at the markers and, thus, coping with the restrictions in the optimization. However, the global diversity at non-marker loci will be low (as stated before) yielding solutions that do not achieve the intended balance between phenotype and diversity (right part of Table 3).

In general, the higher the number of markers used to estimate relationships the higher the *H*_*e*_ retained in the base population. However, the differences in *H*_*e*_ with different marker densities were small. The largest difference occurred in the Mixed scenario under strategy IP (3% higher *H*_*e*_ when using 100,000 markers instead of 10 markers). Improvements in the level of *H*_*e*_ maintained under IC, which should be the most efficient strategy in terms of diversity captured were never larger than 1% (Table 3).

The genetic diversity maintained in the base population under different strategies was very similar across scenarios. Except for some cases with low number of markers, the strategy capturing the highest levels of neutral *H*_*e*_ was IC, because no other factor but diversity was included in the objective and decisions were taken on the genotypes of the individual candidates and not on the mean strain values. The advantage of this strategy compared to sampling equal number of individuals from each strain (strategy E) ranged from 0.4% (in the Selection scenario) to 1.1% (in the Mixed scenario). This result was obtained because the Selection scenario and Mixed scenario present the highest and the lowest degree of homogeneity between the available strains, respectively.

When using the average strain values (i.e., all individuals from the same strain assumed equivalent) levels of diversity obtained under MC were always lower than with IC although, as stated before, differences were small. With increasing number of markers differences diminished and, eventually, disappeared (see Table 3, Stabilizing scenario).

Strategies MP and IP were intended to select individuals with high phenotypic performance but keeping the same level of diversity than E. When using a low number of markers these strategies did not fulfill the restriction (i.e., *H*_*e*_ was lower under MP or IP than under E; see Table 3) as this was introduced in the formulation through the molecular coancestry calculated from the markers but diversity results were obtained from the non-markers genotypes. With the largest panel (i.e., 100,000 SNPs) IP did maintain the same diversity level than E but MP maintained slightly lower values because of the random sampling of individuals within strains.

### Genetic gain and inbreeding from the breeding program

**Figure 1:**
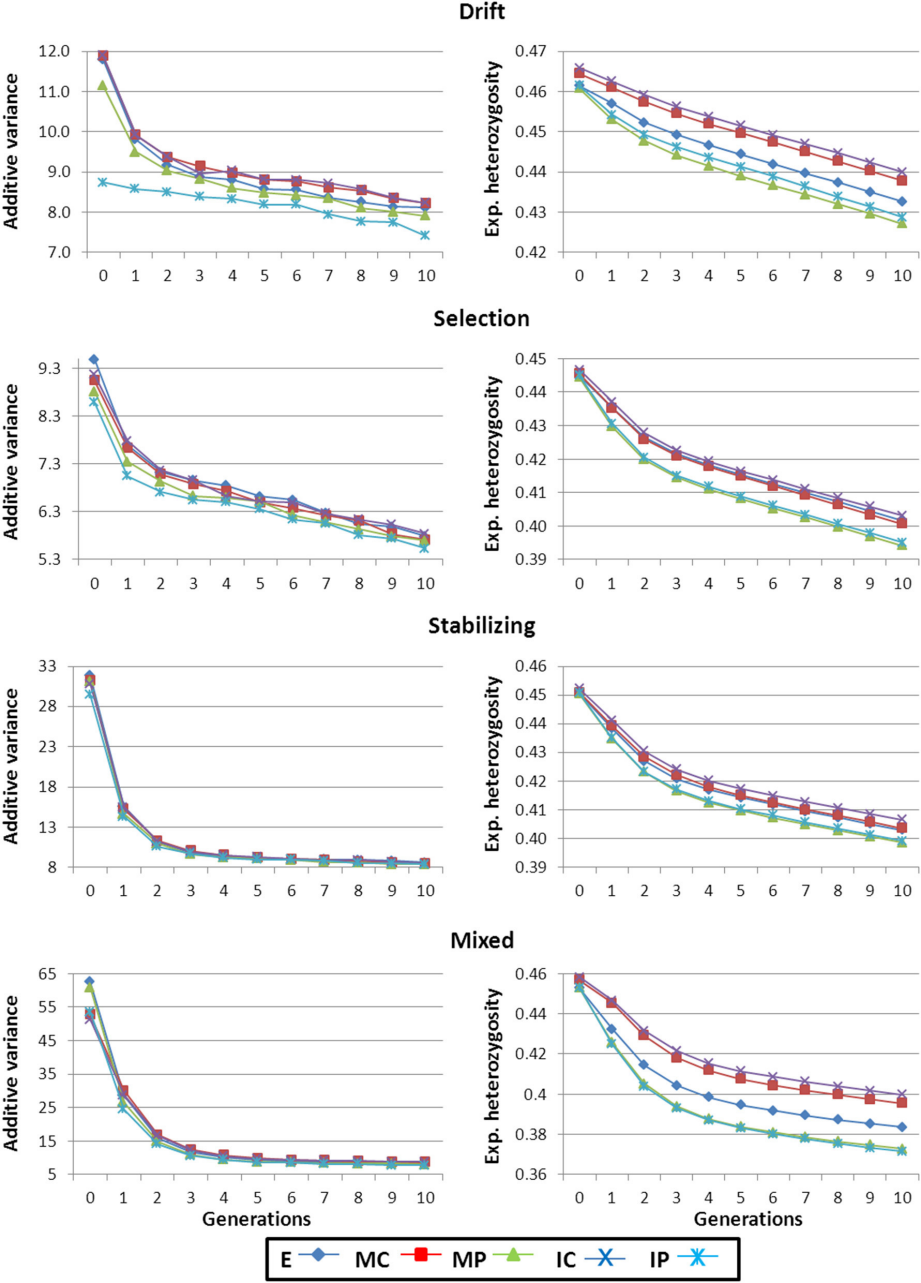
**Additive genetic variance for the selected trait and expected heterozygosity for the non-marker loci along the generations of selection**. Results shown correspond to base populations obtained using 100,000 markers. E, equal numbers from each strain; MC, minimize mean strain coancestry values; MP, maximize mean strain phenotypic value with a restriction on coancestry; IC, minimize individual coancestry; and IP, maximize individual phenotypic value with a restriction on coancestry.

The capability to respond to artificial selection depends on the amount of additive genetic variance (*V*_*A*_) present for the trait. Figure 1 shows *V*_*A*_ along the 10 generations of phenotypic truncation selection for all scenarios and strategies used to construct the base population. The highest initial *V*_*A*_ corresponded to the Mixed scenario as this was the most heterogeneous scenario in terms of types of available strains. The order for the rest of scenarios was Stabilizing, Drift and Selection, following thus the same pattern as that observed for general variability described in the previous section. Within each scenario, patterns of *V*_*A*_ for each strategy were very similar. The highest values were observed for strategy E and the lowest for strategy IP. A large decrease in *V*_*A*_ was observed in early generations in all scenarios due to the Bulmer effect (Falconer and Mackay, 1996).

**Figure 2:**
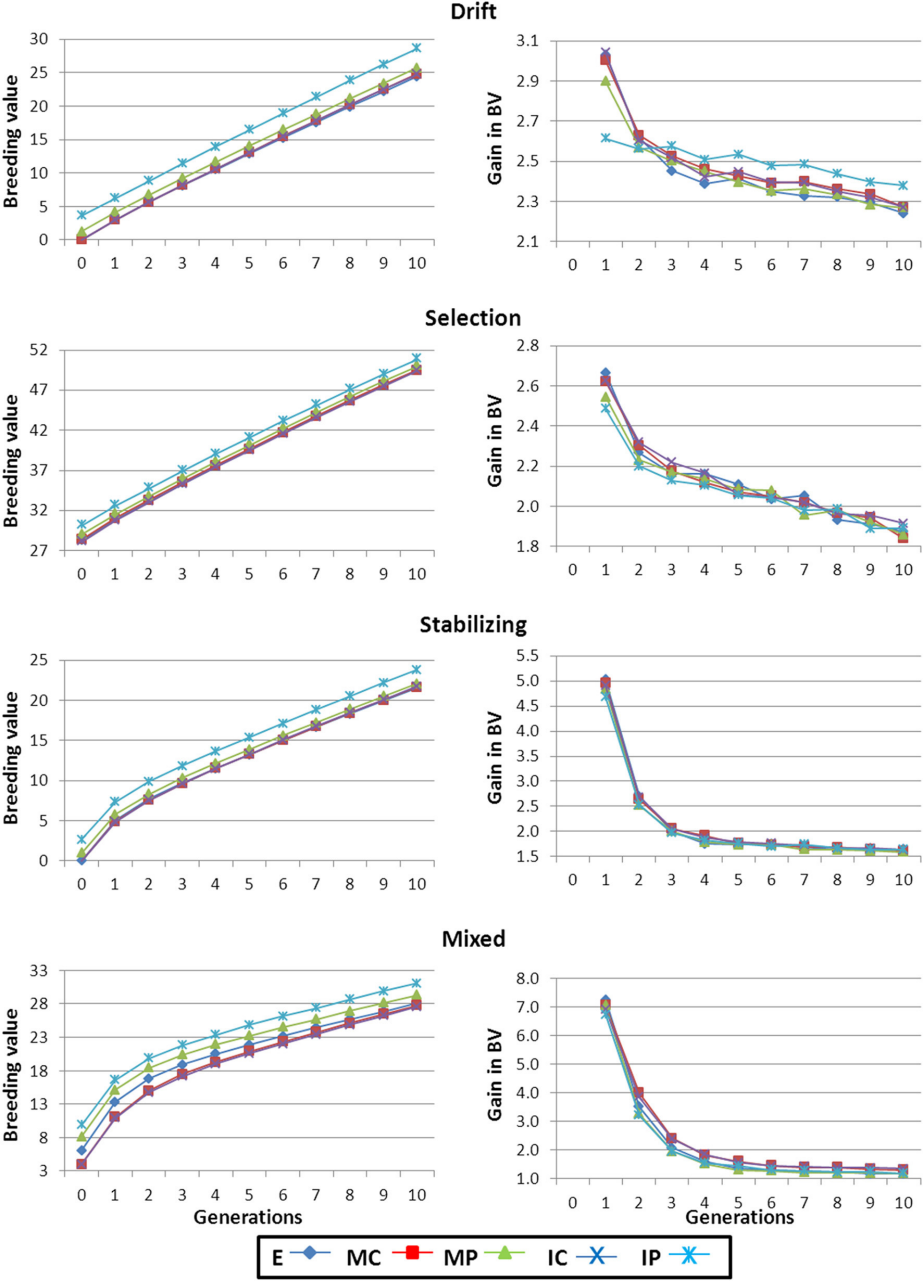
**Average breeding value and gain in breeding value along the generations of selection**. Results shown correspond to base populations obtained using 100,000 markers. E, equal numbers from each strain; MC, minimize mean strain coancestry values; MP, maximize mean strain phenotypic value with a restriction on coancestry; IC, minimize individual coancestry; and IP, maximize individual phenotypic value with a restriction on coancestry.

Higher *V*_*A*_ values in the founders of the breeding program turned into higher initial responses to selection (right panels in Figure 2). After the initial generations of selection, gain increased at a lower rate and, after the 10 generations observed values were, in general, inversely related to the initial *V*_*A*_ (i.e., scenarios with higher initial *V*_*A*_ maintained lower BV gain for the whole period of selection).

The mean breeding value (BV) of the base population was higher for those scenarios including already selected strains (i.e., Selection and Mixed) and close to zero for the other two scenarios (left panels in Figure 2). Irrespective of the differences in the rate of gain for each scenario pointed out before, the rank of scenarios in terms of BV remained the same for the 10 generations of selection.

For a particular scenario, strategy IP always provided the highest BVs (Figure 2), even although generally started from the lowest *V*_*A*_ (Figure 1). When the number of markers used in the creation of the base population was small the advantage (in terms of mean BV) of strategy IP was greater than that presented in Figure 2 (data not shown) due to the higher initial differences with the rest of strategies in trait performance already shown when constructing the base population. But, as discussed before, strategy IP also resulted in lower *H*_*e*_ values (Table 3). In all scenarios, strategies MC and IC performed almost identically for *V*_*A*_, BV and gain, especially when using a large panel of markers (left panel in Figure 1 and both in Figure 2). Strategy E yielded similar results to those from MC and IC except for the Mixed scenario at early generations where the average BV was higher for E (left panels in Figure 2). However, at the end of the 10 generations of selection average BV for E, MC, and IC equalized.

It must be highlighted that the trait under selection was simulated with an additive gene action within and across loci. This is the reason for a continuous decay of *V*_*A*_ for the trait and the corresponding decrease of genetic gain between consecutive generations (Figures 1, 2, respectively). In traits with an important non-additive component the selection process may generate new additive variance which could lead to the maintenance of levels of response to selection larger than expected under a pure additive model.

The Drift scenario started from the highest *H*_*e*_ levels and also showed the lowest rate of loss of diversity along the breeding program (right panels in Figure 1). On the other hand, the fastest decrease in diversity was observed in the Mixed scenario and this was related to the large initial responses obtained under this scenario.

For all scenarios, populations arising from strategies accounting for the phenotypic level of the founders (i.e., MP and IP) lost more *H*_*e*_ during the 10 generation of selection than strategies aiming just at keeping diversity (right panels in Figure 1). This could be due to the fact that in groups of selected individuals the genetic variance for the trait would be more correlated to the global genetic diversity under MP and IP strategies than in the other strategies. Consequently, during the breeding programme the reduction in *V*_*A*_ inherent to the selection process also imply greater reductions in *H*_*e*_ across generations. When initial breeders were chosen based on the genotypes for few markers, populations obtained following IC strategy maintained higher levels of diversity along the generations of selection than when using MC (data not shown) but when a large panel of SNPs was used the performance of both strategies was similar (right panels in Figure 1).

**Figure 3:**
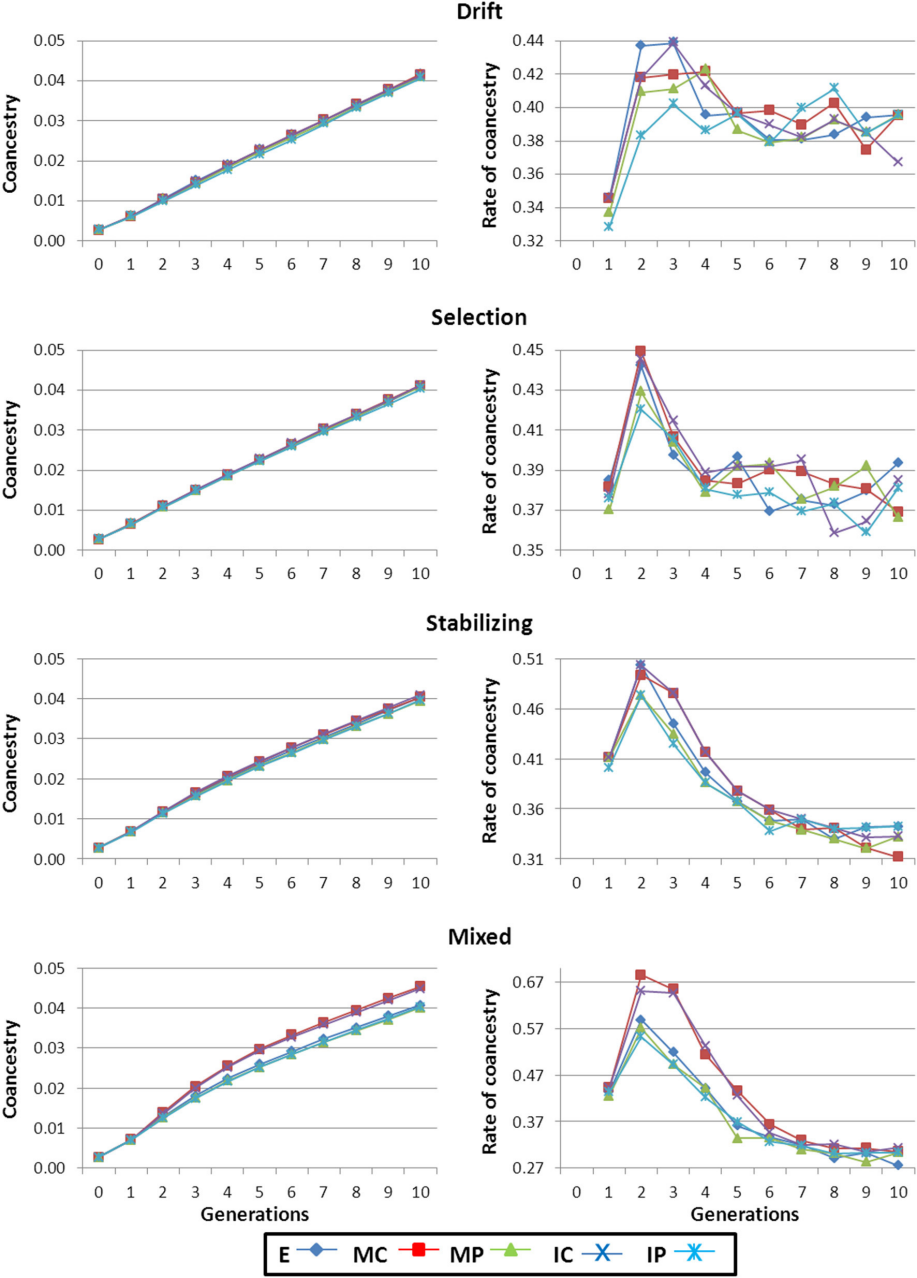
**Average genealogical coancestry coefficient and rate of coancestry along the generations of selection**. Results shown correspond to base populations obtained using 100,000 markers. E, equal numbers from each strain; MC, minimize mean strain coancestry values; MP, maximize mean strain phenotypic value with a restriction on coancestry; IC, minimize individual coancestry; and IP, maximize individual phenotypic value with a restriction on coancestry.

As mating was at random throughout the selection process, average inbreeding (*F*) and coancestry (*f*) coefficients run in parallel, with the expected lag for *F*. Therefore, only results for *f* are shown. Especially for the Mixed scenario (and to a lesser extent for the Stabilizing scenario) *f* was higher for strategies MC and IC than for the rest (left panels in Figure 3). The reason is that strategies MC and IC keep higher numbers of “low performance” individuals whose descendants will not be selected, leading to higher Δ*f* and, thus, to lower *N*_*e*_ than expected. This effect was not detectable in the Selection and Drift scenarios due to the higher homogeneity between strains and individuals for the phenotypic level of the trait.

Irrespective of the scenario, at the beginning of the breeding program there was an increase in Δ*f* (right panels in Figure 3) that was due to the removal of individuals with low genetic BV for the trait. Afterwards, Δ*f* stabilized in the Drift and Selection scenarios around 0.4%. This figure is higher than the expected rate (Δ*f* = 0.25%) for a random selection population of size 200 (i.e., the number of selected individuals each generation; Woolliams and Bijma, 2000) because between-family selection occurs. In the Stabilizing and Mixed scenarios, Δ*f* monotonically decreased reaching levels closer to 0.25%.

## Discussion

The present study has shown that the use of phenotypic information of the candidate strains and the use of genome-wide marker information to infer relationships within and between strains can help to optimize the proportion of individuals to be sampled from each strain when creating base populations for breeding programs in aquaculture species. The advantage of using this information is reflected in the phenotypic and breeding values obtained at the beginning of the program and at the genetic diversity captured by the base population. The advantage remains during the subsequent generations of selection, making the breeding program more profitable. The study has not been designed for a particular species. Instead, we have considered a general genome architecture and a population structure that fit most aquaculture species.

Traditionally, when creating base populations for the establishment of a breeding program, no information was available about the genetic relationships between candidate strains or between individuals within strains. Consequently, the usual strategy was to collect equal number of individuals from as many strains as possible (Holtsmark et al., 2006, 2008a,b). However, the increasing amount of molecular markers developed for aquaculture species provide us with the opportunity of estimating genetic relationships within and between strains and to optimize the contribution of each strain. In this study it has been shown that strains harboring low levels of genetic diversity should contribute less individuals in order to maximize the global diversity of the base population.

In the creation of base populations, one important point to determine beforehand is if the objective is to maximize the genetic variance for a particular trait (i.e., the trait in the breeding goal) or to maintain the highest global diversity. For the former objective, Bennewitz and Meuwissen (2005) showed that the optimal strategy is what they called maximum variance total (MVT), which gives more weight to the variance between strains than within strains. However, although the profitability of the breeding program depends on the performance for the target trait, diversity must be also maintained for other traits that are likely to be included in future breeding objectives and for fitness related traits. Following this logic the methodology should be minimizing the global coancestry which poses the same weight to within and between strains diversity. The latter method is similar to minimizing the long-term inbreeding of the population as demonstrated by Eding and Meuwissen (2001) and was the chosen strategy for the present study. Accordingly, in our results for three of the simulated scenarios, strategy E yielded the highest *V*_*A*_ for the target trait in the base population. The lower levels of *V*_*A*_ observed under strategies MC and IC were due to the fact that the objective was to maximize the global genetic diversity measured as *H*_*e*_ across all the genome. Hayes et al. (2006) compared random with marker-based optimized selection of breeders from a single population of Atlantic salmon in terms of the genetic variance captured for three different traits (growth and two disease traits). They followed an equivalent methodology to strategy IC presented in this study and found higher additive variances in the breeders for the disease resistance traits but a lower variance for growth when optimizing the selection than when breeders were chosen at random. The explanation for these contrasting results was the different genetic architecture of the traits. It must be noticed that the simulated trait in the present study was controlled by a large number of additive loci and had an intermediate heritability typical for growth. This is the reason for similar performance (i.e., highest levels under E than IC strategy) observed in Hayes et al. (2006) for growth and in the present study (at least for three of the simulated scenarios). Another problem for the interpretation of the results in Hayes et al. (2006) is that they employed 237 AFLPs and this number may be not enough for obtaining a high correlation between diversity at markers and at loci controlling growth.

In concordance with the previous considerations, in this study the highest levels of global diversity (*H*_*e*_ measured at the non-marker loci) were captured when optimizing the creation of the base population using individual genotypes (strategy IC) with a large number of SNPs, although differences with the strategy equalizing proportions (strategy E) were small. Surprisingly, scenarios with a limited number of markers (i.e., 100) implied only a loss in *H*_*e*_ of 1% when comparing with results from using large numbers. In any case, when using few markers the *H*_*e*_ maintained under IC was sometimes lower than under the E strategy because of the lack of correlation between diversity at markers and diversity in the rest of the genome.

When individual information was absent (i.e., strategy MC) there was a reduction in the ability to capture diversity respect IC strategy whatever the number of markers used. Notwithstanding, values of *H*_*e*_ obtained when relying on strain averages were less than 2% lower than those observed when individual genotypes of candidates were available. This is an appealing result for cases where the budget is low and no all candidates can be genotyped.

Beyond all considerations about the genetic diversity, we must remember that the short-term profitability of a breeding program depends on actual mean levels of the phenotypic value for the trait of interest (as long as the breeding goal does not change and no fitness troubles arise in the population). The present study has shown that the mean phenotype of the selected individuals should be also accounted for when constructing the base population if that information is available. The loss of profit resulting from including low performance individuals in the base population may be not economically compensated in a reasonable period of time even if the response to selection is high due to a wider genetic variance for the trait. In fact our results showed that superiority of individuals selected under strategy IP last for the 10 generations of selection.

The presented results showed that, when reliable information is available for a fixed set of strains, a compromise solution between diversity and performance can be found when creating base populations. Having as a reference point the strategy randomly sampling the same number of individuals from each strain (strategy E, equivalent to the methodology used in Holtsmark et al., 2006, 2008a,b) up to 7% higher levels of phenotypic performance can be achieved under strategy IP at the same level of global diversity (*H*_*e*_ measured at the non-marker loci) when using a large panel of SNPs to genotype all candidates. Depending on the market value for the increase of one unit of the target trait this could translate into a large economic gain. Moreover, results showed that phenotypic values remain higher during all generations of artificial selection that were simulated from the base population under the IP strategy. Therefore, there was a clear superiority of fishes obtained using this strategy.

It must be realized that giving a large weight to the phenotypic value for a particular trait will have consequences on other correlated traits. Special attention should be paid for traits negatively correlated with the target trait which could be of potential interest. In such situations a useful strategy would be to use an index that includes several traits in the objective function to be maximized or to include an additional restriction in the optimization to ensure a minimum acceptable phenotypic level for the secondary traits.

It must be stressed that genotyping for a limited number of markers may give undesirable results because diversity at those markers will be loosely related to diversity at non-marker loci and to diversity at loci controlling the trait. Consequently, individuals with a high phenotypic performance may actually maintain little global diversity but still exhibit by chance high levels of diversity at the markers. Our results show that, when relaying in few markers, lower *H*_*e*_ levels were found when optimizing contributions using the IP strategy than using the E strategy even when a restriction was imposed to keep the same level of diversity (Table 3). However, results from our simulations suggest that, although it will depend on the particular characteristics of the species under management and the genetic architecture of the available strains, about 1000 SNPs could be enough to efficiently create base populations in aquaculture as no relevant improvements are obtained by increasing further the number of markers.

For all scenarios, strategies accounting for the phenotypic level of the founders (i.e., MP and IP) started the selection program from lower values of *V*_*A*_ for the target trait. This fact did not preclude these strategies to maintain the initial advantage in performance during the 10 generations of selection.

Results of this study suggest that if “healthy” commercial strains (i.e., where diversity is not exhausted and no problems of inbreeding exist) are available they should be used to form the base population because they provide a higher starting performance level. This is even clearer when the objective is to complement the breeders' population in an ongoing selection program. Contrarily, the use of wild adapted strains with low performance would be only recommended if we suspect that unique information for other traits of interest is present in them. This could be the situation for strains naturally resistant to a particular disease. Otherwise, the general diversity that they could provide will not compensate for the lower trait phenotypic mean.

The small differences in *H*_*e*_ observed under different strategies in our simulations could be due to the large number of individuals selected to form the base population (200) making strategy E to perform so well that the other strategies have difficulties in improving *H*_*e*_. In an extra scenario run with a smaller set of candidates (only four strains with 20 individuals each), harboring lower levels of diversity (mutation-drift equilibrium reached for a population of 100 individuals) and selecting a lower number of breeders (24), strategies implying optimized proportions showed still only slightly greater advantages over strategy E (2% increase). In any case, the differences observed in phenotypic values make worthy to optimize the construction of base populations in aquaculture and levels of diversity should be also accounted for in that task to get an appropriate balance.

Another advantage of using molecular information, beyond balancing phenotypic values and diversity when optimizing the construction of a base population, is that it provides us with the possibility of estimating the actual relationships between breeders in the base population itself. These relationships can be used for calculating EBVs through BLUP methodology and also for controlling the rate of inbreeding through Optimal Contribution strategies. Holtsmark et al. (2008b) studied the effects on the performance of the breeding program across generations of assuming unrelated and non-inbred founders when they are not. They concluded that an incorrect estimation of the relationships between and within strains and individuals leads to sub-optimal use of subpopulations with an increased risk of loss of alleles of direct and strategic relevance to the breeding program.

If genotypes for dense panel of markers and phenotypes are available for the same individuals (the candidates to be part of the base population or related individuals), the additive effect of each SNP can be calculated in the same way as in the Genomic Selection methodology (Meuwissen et al., 2001). Thus, genomic value of candidates can be calculated and used to take decisions instead of their phenotypic value.

In the present study the selected trait was simulated with an additive gene action both within and across loci. However, traits with commercial interest may have an important dominant component. Dominant effects can be also estimated and then used to design the mating scheme between breeders, at least to form the families from which the selection program will start. This way, the effects of heterosis can be accounted for and extra responses can be obtained in the first round of selection (Toro and Varona, 2010).

In short life species, where individuals may be not reproductively active by the time their phenotypic records and genotypes are available, it would be difficult to implement strategies based on individual information (i.e., IC and IP). However, even in such situations strain information can be used to optimize the creation of the base population, as proved in the present study. The impossibility of controlling the specific matings with mass spawning species does not interfere with the optimization of the base population either. Phenotypic levels and diversity can be optimized at the start of the program although responses in subsequent generations of selection may differ to those shown here given that selected breeders would need to be mated in groups.

In livestock terrestrial species, breeding programs have been running for many years and, thus, it is not very likely that there is a need for creating new base populations. Notwithstanding, our conclusions go beyond the scope of creating base populations. For instance, they may help to take decisions when creating a gene bank for any species. When the aim of such a bank is to store the genetic diversity from the available strains, the same strategies can be applied as when creating the base population of breeders. When, in the future, the stored material will be used for creating a live population or for complementing a breeding program the same results and consequences than those presented here are expected. If the phenotypic values of the candidates are not taken into consideration when determining the sampling scheme the starting population will show low levels for the trait of interest. Finally, the methodology presented in this study is also useful when the objective is to create a “core” live population within an ex-situ conservation program that aim at collecting the genetic diversity that exist in all available populations. This scenario is common for local breeds of livestock species.

### Conflict of interest statement

The authors declare that the research was conducted in the absence of any commercial or financial relationships that could be construed as a potential conflict of interest.
